# Mid-term clinical outcomes of totally endoscopic repair for mitral regurgitation in Barlow’s disease

**DOI:** 10.1186/s13019-024-02705-y

**Published:** 2024-04-16

**Authors:** Lin Zhang, Huimin Cui, Hong Shen, Dong Li, Lianggang Li, Hua Shen, Shengli Jiang

**Affiliations:** Department of Cardiovascular Surgery, the First Medical Center of Chinese PLA General Hospital, 28 Fuxing Road, Beijing, 100853 China

**Keywords:** Barlow’s disease, Totally endoscopic, Mitral valve repair, Mitral regurgitation

## Abstract

**Objective:**

This study aimed to confirm the safety and feasibility of totally endoscopic repair for mitral regurgitation (MR) in Barlow’s disease.

**Methods:**

From June 2018 to December 2022, 21 consecutive Barlow’s disease patients (aged 33 ± 12 years; 57.1% male) underwent totally endoscopic mitral valve (MV) repair with leaflets folding, multiple artificial chordae implantation and ring annuloplasty. The safety and feasibility of this technique was evaluated by its mid-term clinical outcomes.

**Results:**

There was no operative death or complications. The mean cardiopulmonary bypass (CPB) time was 190 ± 41 (128–267) min, and the aortic cross-clamp time was 145 ± 32 (66–200) min. The average number of artificial chordae implantation was 2.9 ± 0.7 (1–4) pairs. The mean MV coaptation length was 1.4 ± 0.3 (0.8–1.8) cm, and the median transvalvular gradient was 1 [interquartile range (IQR), 1–2] mmHg. During a median follow-up time of 24 (IQR, 10–38) months, all patients showed persistent effective valve function with no significant MR or systolic anterior motion.

**Conclusions:**

Totally endoscopic repair was a safe, effective, and reproducible procedure with satisfied mid-term clinical outcomes for MR in Barlow’s disease. However, further randomized and long-term follow-up studies were warranted to determine its clinical effects.

## Introduction

Barlow’s disease is the most frequent degenerative valvular disease with a prevalence of 4–5% in the general population [1]. It is characterized by excess thickened leaflet tissue, a severely dilated annulus, with or without leaflet prolapse, manifesting typically in young patients [2]. Mitral valve (MV) repair for this myxomatous disease, which accounts for up to 30% of MV repair surgery [3], is a particularly challenging procedure for its complex form of pathology, which involves leaflets, annulus, chordae, and papillary muscle motion. Good repair requires understanding of these changes [4].

Different from the conventional MV surgery through a median sternotomy, minimally invasive cardiac surgery, which is dedicated to minimizing surgical trauma, accelerating postoperative recovery and improving cosmetic effect of incision, has been developed rapidly during the past decades [5]. The adoption of endoscopy in cardiovascular surgery has been the trend of the minimally invasive cardiac procedure. However, there are only few literatures about totally endoscopic MV repair in Barlow’s disease. As one of the medical centers that perform totally endoscopic valvular surgery as routine, we share our experience and report our mid-term clinical outcomes of totally endoscopic MV repair for MR in Barlow’s disease, no matter with multisegment involvement or limited leaflets prolapse. The safety and feasibility of this technique is evaluated by its mid-term clinical outcomes.

## Materials and methods

### Patients

From June 2018 to December 2022, a total of 406 consecutive patients underwent totally endoscopic MV repair in Chinese PLA General Hospital by the same experienced surgeon. Among them, 21 cases (5.17%) were identified as Barlow’s disease. There were 12 males and 9 females with an average age of 33 ± 12 (18–63) years. A distinct late systolic murmur and non-ejection systolic click was audible in the mitral auscultation area in all patients during the physical examination of admission. The severity of mitral regurgitation (MR) was assessed by preoperative transthoracic echocardiography (TTE), and it was classified into 0 (none or trivial), 1+ (mild), 2+ (moderate), 3+ (moderate-severe), or 4+ (severe). 14 patients were in severe MR, 6 in moderate-severe and 1 in moderate. 14 patients (66.7%) presented with localized leaflet prolapse, among which chordae rupture was observed in 5 cases (23.8%) in P2 area. The remaining 7 (33.3%) presented with multisegment involvement causing mainly central jet. 18 patients (85.7%) showed MR related symptoms and their New York Heart Association (NYHA) functional class was ranging from II to IV. 17 patients (81.0%) presented with left atrial enlargement (> 35 mm), and the mean left atrial internal diameter was 39.9 ± 4.4 (30–46) mm. The mean left ventricular end diastolic dimension (LVDD) was 49.9 ± 5.6 (40–61) mm, and the mean left ventricular ejection fraction (LVEF) was 65.3 ± 7.2 (52–79) %. Electrocardiogram suggested 20 patients were in sinus rhythm (SR) and 1 in frequent premature ventricular contraction (FPVC). Some patients received coronary angiography before operation to exclude concomitant coronary artery disease. Clinical characteristics of the patients were present in Table 1.

**Table 1:** Preoperative characteristics

Variable	Value
Male/Female	12/9
Age (years)	33 ± 12
Body mass index (Kg/m^2^)	22.6 ± 5.6
Frequent premature ventricular contraction	1
New York Heart Association functional class	
I	3
II	16
III	1
IV	1
**Transthoracic echocardiography**	
Left atrial internal diameter (mm)	39.9 ± 4.4
Left ventricular end diastole diameter (mm)	49.9 ± 5.6
Ejection fraction (%)	65.3 ± 7.2
Mitral regurgitation	
4+	14
3+	6
2+	1
Mitral valve leaflet prolapse	14
Mitral valve chordae rupture	5

### Surgical technique

After the induction of general anesthesia, a left-sided double-lumen endotracheal tube was placed to allow for single-lung ventilation. Placement of defibrillator pads across the chest wall was routine, as access to the ventricles was limited. The patients were positioned supine all the way with the right hemithorax elevated to 30° with a small pillow placed inferior to the scapula to fully open up the axillary space. The right arm was tucked at the side lower than chest to improve access to the anterior axillary line. A transesophageal echocardiography (TEE) probe was then placed to evaluate the MV function during operation.

The cardiopulmonary bypass (CPB) was instituted via right internal jugular vein, femoral arterial and femoral vein. A right lateral incision (about 3.5 cm in length) was located in the 4th intercostal space on the right midclavicular line as the main operating hole, through which the cardioplegia irrigation tube was also passed. The endoscopy was inserted through the right lateral 4th intercostal space on the anterior axillary line, while the left cardiac drainage tube and Chitwood aortic clamp were punctured from the 5th intercostal space on the mid-axillary line. CO2 insufflation was routinely used. The MV was routinely visualized through the left atrium incision running parallelly to the interatrial groove. A left atrial retractor penetrating from the 3rd or 4th intercostal space on the right side of the parasternal bone was used to rise the left atrial free wall for a full exposure of MV.

The MV repair was begun with artificial chordae implantation in the P2, A2, and P1 area, respectively, sometimes even in the A1 area; then continued with two or three magic stitches to advance the posterior commissure medially to accomplish the leaflets folding; and ended with a annuloplasty ring placement. We adopted a special method to implant the artificial chordae, by which the artificial chordae was interlocked into an adjustable loose knot. After the papillary muscle was sutured with 4 − 0 Gore-Tex, the suture was threaded from the left ventricular surface of the MV to the left atrial surface 6–8 mm away from the free edge, and once again 3–4 mm away in the same direction. Once the annuloplasty ring was lowered into the position, a preliminary water sealing test before tying the sutures would be taken to guarantee there was no residual regurgitation.

### MV repair assessment

TEE post-CPB was repeated in all patients to assess the efficacy of the MV repair including the structure and function, and more than mild residual MR would result in a repeat MV repair or replacement procedure. All patients were routinely examined by TTE one week after surgery.

### Follow-up

TTE was performed before discharge, 3 months later and then annually after surgery to assess the postoperative condition of patients. Follow-up was conducted by outpatient visits and telephone. The data mainly included results of TTE, recurrence rate of moderate or severe (3 + or 4+) MR, cardiac related hospital re-admission and the quality of life. The latest follow-up data was collected between November 2022 and March 2023.

### Statistical analysis

SPSS23.0 Software (SPSS, Inc., Chicago, IL, USA) was applied to analyze the data. Continuous variables were tested the for the normality. Afterwards, normally distributed continuous data was expressed by the mean and standard deviation and not normally distributed data by median and range [interquartile range (IQR)]. Categorical variables were presented as frequencies and percentages. A significant difference for hypothesis testing was set at *P* < 0.05.

### Ethics statement

The study was approved by the Ethics Committee of Chinese PLA General Hospital. Written informed consent was obtained from all patients before surgery.

## Results

### Intraoperative data

All patients were confirmed with moderate to severe or severe MR by intraoperative TEE after anesthesia. Typical characteristics of Barlow’s disease were detected under direct vision during the operation. Multiple artificial chordae were implanted according to the different locations of the prolapsed leaflet with an average of 2.9 ± 0.7 (1–4) pairs. Leaflets folding with internal commissure suture was performed in all patients. Interrupted sutures were placed along the annulus to implant the annuloplasty ring, including the latest 11 cases with Physio II ring [Carpentier-Edwards, mean size: 34.2 ± 1.0 (32–36) mm] and previous 10 with Cosgrove (Edwards, all in 34 mm). The mean size of the annuloplasty ring was 34.1 ± 0.8 (32–36) mm. The mean CPB time was 190 ± 41 (128–267) min, mean aortic cross-clamp time was 145 ± 32 (66–200) min, and the duration of surgery was 289 ± 48 (220–420) min. The results of immediate intraoperative MV repair were no MR in 18 cases and mild in 3. The mean MV coaptation length was 1.4 ± 0.3 (0.8–1.8) cm, and the median transvalvular gradient was 1 (IQR, 1–2) mmHg. There was no operative death or related complications. The mean postoperative mechanical ventilation time was 8.7 ± 3.6 (4.2–13.5) h, intensive care unit (ICU) stay time was 18 ± 5 (10–26) h, postoperative drainage was 118 ± 54 (20–280) ml in the first 24 h. Details of surgery were present in Table 2.

**Table 2:** Operative and postoperative data

Variable	Value
**Operative**	
Cardiopulmonary bypass time (min)	190 ± 41
Aortic cross-clamp time (min)	145 ± 32
Total duration of surgery (min)	289 ± 48
Artificial chordae implantation (pair/case)	2.9 ± 0.7
Mitral valve coaptation length (cm)	1.4 ± 0.3
Transvalvular pressure gradient (mmHg)	1 (IQR, 1–2)
Immediate intraoperative mitral regurgitation after repair	
0	18
1+	3
Ring type	
Cosgrove-Edwards	10
Carpentier-Edwards Physio II	11
Ring size (mm)	
Cosgrove	34
Physio II	34.2 ± 1.0
**Postoperative**	
Postoperative mechanical ventilation time (h)	8.7 ± 3.6
Intensive care unit stay time (h)	18 ± 5
Postoperative hospital stay (d)	5.2 ± 0.6
Postoperative drainage in the first 24 h (ml)	118 ± 54
**Follow-up**	
Median follow-up time (months)	24 (IQR, 10–38)
Mitral regurgitation	
0	11
1+	10
Systolic anterior motion	0
New York Heart Association functional class during this follow-up interval	
I	17
II	4

### Follow-up data

All patients had TTE as part of their follow-up, and the median follow-up time was 24 (IQR, 10–38) months. 10 patients exhibited asymptomatic mild MR, and none of the patients had recurrent moderate or severe MR or systolic anterior motion (SAM). The NYHA class revealed 17 patients (81.0%) were asymptomatic (NYHA I), and 4 in NYHA class II. The follow-up data was shown in Table 2. The mean left atrial internal diameter, LVDD and LVEF was 33.6 ± 3.9 (25–40) mm, 46.0 ± 2.9 (39–51) mm and 64.0 ± 5.5 (54–72) %, respectively. The comparison between preoperative and the latest follow-up data of TTE and NYHA class was shown in Table 3. Functional classification improved, as NYHA status changed from the preoperative mean of 2.0 ± 0.6 to 1.2 ± 0.4 during this follow-up interval (*P* < 0.05), with similar change in left atrial internal diameter. Significant differences were observed in NYHA class and left atrial internal diameter.

**Table 3:** The comparison between preoperative and the latest follow-up data of transthoracic echocardiography and New York Heart Association functional class

Variable	Preoperative (21 Patients)	Postoperative (21 Patients)	p Value
Left ventricular end diastolic dimension (mm)	49.9 ± 5.6	46.1 ± 2.9	0.197
Left atrial internal diameter (mm)	39.9 ± 4.5	33.6 ± 3.9	0.016^a^
Ejection fraction (%)	65.3 ± 7.2	64.1 ± 5.5	0.632
Transvalvular pressure gradient (mmHg)	21.9 ± 3.4	1 (IQR, 1–2)	0.380
New York Heart Association functional class	2.0 ± 0.6	1.2 ± 0.4	0.005^a^

^a^*p* < 0.05

## Discussion

In 1996, Carpentier et al. performed the first successful minimally invasive endoscopic MV repair [6]. Since then, endoscopic MV repair procedure has gradually developed. Even with complex valvular disease, totally endoscopic MV repair can achieve satisfactory results due to its appealing advantages [7]. The survival and durability of MV repair for prolapse is superior to the replacement, and it has become the gold standard for the treatment of MR [8, 9]. Based on surgical technique advancement and improved surgical outcomes, the American College of Cardiology (ACC)/American Heart Association (AHA) guidelines for valvular heart disease have recommended more MV repair rather than replacement [10]. However, due to the higher recurrence and reoperation rate of Barlow’s disease [11–13], MV repair in Barlow’s disease remains the largest challenge even for an experienced cardiac surgeon [14].

It has been reported in the literature that the optimal way to reconstruct the MV in Barlow’s disease is to correct both the billowing leaflet and the prolapse of leaflet [2]. The Carpentier quadrangular resection, with or without concomitant sliding plasty, and remolding annuloplasty are considered the standard surgical techniques to repair Barlow’s disease [15]. However, the application of minimally invasive technology in cardiac surgery has greatly changed the surgical approaches of this operation. From June 2018, we have taken our full advantage of technical superiority to perform totally endoscopic MV repair for MR in Barlow’s disease, and achieved excellent mid-term clinical outcomes. The following are discussions about surgical techniques and follow-up.

### Multiple pairs of artificial chordae implantation

Leaflet prolapse is the main cause of significant regurgitation. Correction of the prolapsed leaflet is the key to a good long-term result of MV repair [4]. The technique of MV repair with artificial chordae in Barlow’s disease, which can avoid leaflet resection and increase the area of leaflet coaptation, is an effective method for resolving leaflet prolapse, promising a good long-term follow-up.

In our series, 14 patients (66.7%) had localized leaflet prolapse, among which chordae rupture was observed in 5 cases (23.8%) in P2 area. We located the leaflet prolapse site according to the intraoperative TEE and water sealing test together with preoperative TTE. The P2 area was considered to be the most prone to prolapse as it suffered the greatest pressure during systole [16], thus we implanted the artificial chordae in P2 and A2 area as routine. Sometimes, we even implanted artificial chordae in P1 and A1 area depending on the severity of regurgitation. Multiple pairs of artificial chordae implantation could effectively lower the height of the posterior leaflet and form sufficient A2/P2 height ratio, which could effectively correct the leaflet prolapse and billowing. The special method, by which the artificial chordae was interlocked into an adjustable loose knot, guaranteed the length of artificial chordae was adjustable and ensured that the slipknot was unchangeable during knotting. We never measured the length of the artificial chordae deliberately, which was adjusted according to the experience of the surgeon and the results of the water injection experiment after folding the posterior commissure and remolding annuloplasty. In this study, the average MV leaflet coaptation was 1.4 ± 0.3 cm with no SAM in any case, which fully showed the advantages of artificial chorade technique. We believed multiple artificial chorade technique was the key to the MV repair of Barlow’s disease, and also the key to ensure the long-term effect of the surgery.

### Correction of internal commissure

For those trained in the Carpentier repair techniques, the concept of “excessive leaflet size” to be corrected by leaflet resection is a critical consideration [4]. However, the excision of excess valve tissue and the suturing of the P1 and P3 segments can restrict leaflet motion and it is very challenging for cardiac surgeon. Fortunately, the concept that resection of the “excessive leaflet” in Barlow’s disease is not mandatory has been widely accepted by cardiac surgeons in recent years.

Although the implantation of artificial chordae can pull the mitral leaflet that prolapse into the left atrium back into the left ventricle, the billowing change leaflet is still exist. Mungara, C et al. reported that due to the large size of the mitral orifice in Barlow’s disease, although it was not the optimal procedure for impeding normal function of commissure, correction of limited internal commissure prolapse with two or three uniform sutures across the commissure was a useful supplement to the treatment of complex Barlow’s disease with secondary lesions [17]. Given the specificity of the totally endoscopic procedure, we often used relatively simple techniques, and the posterior commissure was usually advanced medially by two or three magic stitches. In this way, the excessive leaflet tissue was diminished and/or the commissural prolapse was corrected without compromising valve function for a large orifice of Barlow’s valve.

### Ring annuloplasty and selection

Ring annuloplasty is considered as an important auxiliary means for valve repair and an important guarantee for long-term good results of MV repair [17]. In 1987, Levine et al. first described the three-dimensional saddle shape of the mitral annulus [18]. Restoring the normal shape and size of the annulus by using the annuloplasty ring during systole is a prerequisite for normal MV close. The design concept of Physio II ring is based on the MV working mechanism. Jouan et al. suggested using of large size Physio II annuloplasty ring in MV repair and reconstruction in Barlow’s patients [15]. In this study, the latest 11 patients were implanted with Physio II ring with a mean size of 34.2 ± 1.0 (32–36) mm. The Saddle-shaped Physio II forming ring is consistent with the normal physiological state conformation of MV closure. Although this semi-rigid forming ring loses the important function of the annulus relaxation, the Physio II ring can fix the dilatated annulus in the systolic state to ensure the most suitable surface of the MV coaptation. Fixing the dilated annulus in systolic state can reduce the tension of excessive leaflet and chordae, reduce the billowing of leaflet and increase the area of leaflet coaptation, and prevent further dilation of the annulus.

Ben Zekry et al. reported that simple annuloplasty in Barlow’s disease with a preoperative diagnosis of moderate to severe or severe MR could also achieve excellent long-term results and good reproducibility [19]. However, we believed that in the absence of an effective sub-valvular traction structure, the leaflets that had recovered into the left ventricular cavity could still be pushed upward into the left atrium by systolic blood flow, leading to progressive leaflet billowing and chordae lengthening until new leaflet prolapse or chordae rupture occurred. Moreover, the height of the posterior leaflet was not reduced with only annuloplasty, and the increase of leaflet surface tension, especially in the P2 portion, could lead to chordae rupture according to Laplace’s law. Thus, considering the complex form of pathology in Barlow’s disease, which involves leaflets, annulus, chordae, and papillary muscle motion, we routinely adopted leaflets folding, multiple artificial chordae implantation and ring annuloplasty to accomplish the totally endoscopic MV repair. Our mid-term follow-up data suggested that the technique was safe, effective and repeatable with excellent clinical outcomes.

## Conclusions

Totally endoscopic repair was a safe, effective, and reproducible procedure with satisfied mid-term clinical outcomes for MR in Barlow’s disease. However, further randomized and long-term follow-up studies were warranted to determine its clinical effects.

## Limitations of the study

The limitation of this study related to the fact that this was non-randomized, retrospectively analyzed data from a small number of patients with short follow-up period in a single center experience. Further randomized and long-term follow-up studies were warranted to determine the clinical effects of the totally endoscopic repair for MR in Barlow’s disease.

## Data Availability

Supporting data are available through the corresponding author on reasonable request.

## Abbreviations

MR: mitral regurgitation
MV: mitral valve
CPB: cardiopulmonary bypass
IQR: interquartile range
TTE: transthoracic echocardiography
NYHA: New York Heart Association
LVDD: left ventricular end diastolic dimension
LVEF: left ventricular ejection fraction
SR: sinus rhythm
FPVC: frequent premature ventricular contraction
TEE: transesophageal echocardiography
ICU: intensive care unit
SAM: systolic anterior motion
ACC: American College of Cardiology
AHA: American Heart Association
